# Endoscopic thulium-fiber laser deroofing and dual stenting for adult duplex ureterocele with preserved upper renal moiety: a case report

**DOI:** 10.1097/MS9.0000000000003704

**Published:** 2025-08-08

**Authors:** Sai Nikhitha Malapati, Ashok Adhikari, Venkata Akhil Makarla, Sai Geethika Malapati, Krishnam Raju Dema, Apsha Shrestha

**Affiliations:** a Kamineni Academy of Medical Sciences and Research Center, Hyderabad, India; b Universal College of Medical Sciences, Bhairahawa, Nepal; c Mamata Medical College, Khammam, India; d Department of Pediatrics, ESIC Medical College & PGIMSR & Model Hospital, Rajaji Nagar, India

**Keywords:** case report, dual DJ stent, duplex kidney, preserved function, thulium fiber laser, ureterocele

## Abstract

**Background:**

Adult duplex kidney with ureterocele is rarely diagnosed, especially when the upper moiety retains function. While pediatric laser decompression is established, the use of thulium fiber laser (TFL) with dual DJ stenting in adult duplex ureteroceles remains rare.

**Case Presentation:**

A 23-year-old female presented in a tertiary care hospital with intermittent right flank pain and recurrent UTIs for 3 months. Imaging revealed a right-sided duplex system with an intravesical ureterocele and functioning upper moiety. TFL deroofing was performed, with placement of dual DJ stents. Operative time was 40 minutes with negligible bleeding. Recovery was uneventful, and stents were removed at 6 weeks. At 6 months, imaging confirmed resolved hydronephrosis and preserved function; the patient remained asymptomatic at 12-month tele-follow-up.

**Clinical Discussion:**

Ureteroceles result from ureteric bud maldevelopment and are often associated with duplex systems per the Weigert-Meyer law. Adult presentations may include recurrent UTIs, hematuria, or flank pain. Imaging modalities such as ultrasonography, CT urography, and renography are essential for diagnosis and surgical planning that prioritizes decompression and nephron preservation. Traditional endoscopic methods, including cold-knife incision and Holmium:YAG laser, risk reflux and tissue damage. TFL offers precise, shallow ablation with better hemostasis and reduced complications, though its adult use is underreported. This case represents the first documented TFL deroofing with dual DJ stenting in adult duplex ureterocele, showing excellent anatomical and functional outcomes, suggesting TFL’s potential as a safe, effective option pending larger studies.

**Conclusion:**

TFL with dual DJ stenting offers a precise, nephron-preserving, and effective approach for adult ureterocele management.

## Background

Ureterocele is a consequence of incomplete dissolution of the embryonic Chwalla membrane or failed canalization of the distal ureteric bud. It’s commonly diagnosed neonatally through routine screening with a marked female predilection and a subtle bias for the left side^[1]^. Its occurrence in adults on the right side, especially in association with complete duplex renal systems with preserved function, remains exceedingly rare^[1,2]^. The adult-onset discovery of a ureterocele in conjunction with a functioning upper renal moiety in a duplicated system is not just rare; it represents a true clinical anomaly with profound implications for nephron-sparing management^[2]^.

**HIGHLIGHTS**

- Rare adult duplex-system ureterocele successfully treated with thulium fiber laser (TFL) and dual DJ stenting.
- Dual DJ stenting safeguarded both moieties, ensuring optimal drainage and recovery.
- Postoperative follow-up showed preserved renal function with no reflux or hydronephrosis.
- First adult case reported from India managed using TFL and dual DJ stents approach, extending pediatric techniques to adults.

Historically, endoscopic management of ureteroceles has relied on techniques such as cold-knife incision, monopolar diathermy, or Holmium: YAG laser ablation. However, these modalities present well-documented limitations, including significant risks of postoperative vesicoureteral reflux (VUR), thermal injury to adjacent tissues, hemorrhage, incomplete decompression, and recurrent symptoms^[3,4]^. In recent years, the Thulium Fiber Laser (TFL) has emerged as a technological breakthrough in endourological surgery, achieving exceptionally shallow penetration (~0.2 mm), continuous-wave energy delivery, and utilizing ultra-thin fibers (≤150 µm), permitting unparalleled precision in delicate surgical fields^[5]^. Clinical studies, particularly in pediatric populations, have reported treatment success rates as high as 92%, with drastically reduced rates of postoperative reflux, while in the adult urologic landscape, robust evidence for TFL use in ureterocele management remains scarce, confined to anecdotal case reports^[6–8]^. We present a case of a 23-year-old woman diagnosed with a right duplex collecting system and an intravesical ureterocele with preserved function. Treatment included TFL and dual ureter DJ stenting for 6 weeks, with regular follow-up after 6 months and a teleconsultation follow-up at 12 months. This case report has been reported in line with the SCARE 2025 guidelines^[9]^.

## Case presentation

**Figure 1.**
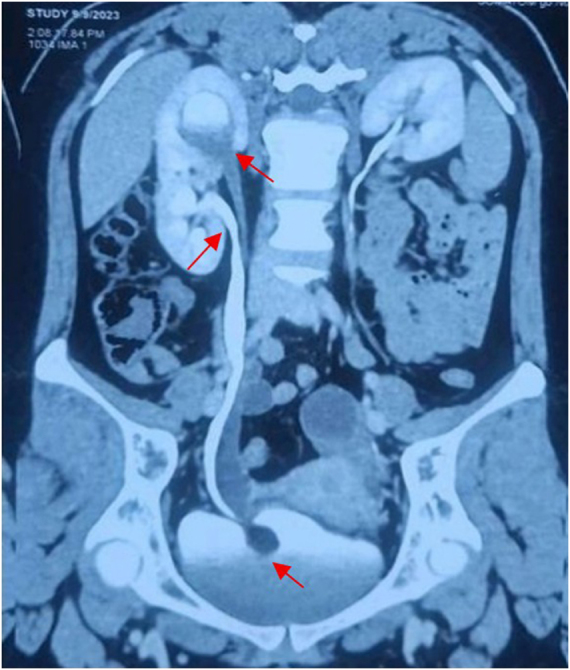
CT-urography depicting the right duplex with “cobra-head” appearing intravesical ureterocele (with red arrows).

A 23-year-old nulliparous South-Indian female presented to our tertiary care hospital outpatient department with a three-month history of intermittent right flank pain worsened by physical activity, with no hematuria or lithuria. Her past medical history was remarkable only for two culture-positive urinary tract infections (UTIs). The vital signs were normal, and upon physical examination, no abnormalities were detected except for mild costovertebral angle tenderness. The renal function tests were normal: creatinine (0.8 mg/dL) and Glomerular filtration rate (GFR, >90 mL/min/1.73 m^2^), while the urinalysis showed pyuria and cultured E. coli sensitive to nitrofurantoin. A renal ultrasound showed a Right duplex system with moderate upper moiety hydronephrosis. The CT-urography (split-bolus) confirmed the diagnosis along with intravesical 20 × 20 mm ureterocele with “cobra-head appearance (Fig. 1).

We ordered a Technetium-99 m DTPA renogram to assess the kidney function, which showed right kidney function at 52.7% with upper moiety contributing 30% and delayed drainage (T½, half-life >20 min). A multidisciplinary team judged the upper moiety salvageable and recommended TFL decompression over heminephrectomy.

## Operative technique and immediate outcome

**Figure 2.**
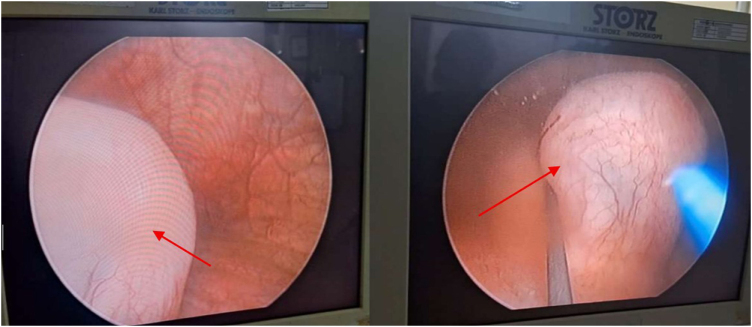
Cystoscopy revealing translucent cyst at right trigone (red arrows).

**Figure 3.**
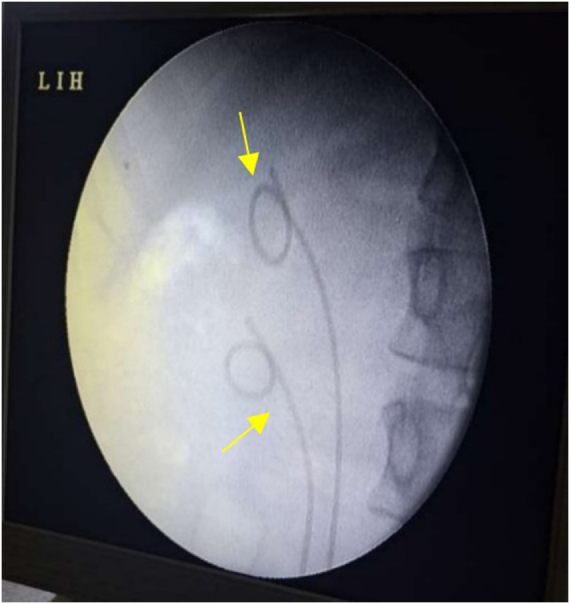
Dual DJ stents placement post procedure (yellow arrows).

Under general anesthesia in lithotomy position, rigid cystoscopy revealed a translucent cyst at the right trigone (Fig. 2). A 550-µm TFL fiber (RevoLix® DUO) at 15 W, 1.5 J, 10 Hz was used for a 1.5 cm longitudinal incision. Clear urine drained, and hemostasis was immediate due to TFL’s superficial effect. Retrograde pyelography confirmed duplex anatomy, and two DJ stents were placed: 4.5 Fr (upper moiety) and 4.0 Fr (lower moiety) (Fig. 3). Total laser time: 3 minutes; total procedure: 40 minutes; negligible blood loss.

## Postoperative course and follow-up

Ambulation at 6 hours, diet resumed by 8 hours, discharge on day 1 with nitrofurantoin prophylaxis; Foley removed at 12 hours. Only transient irritative symptoms (Clavien–Dindo I).

- 6-week follow-up: Stents removed; USG showed resolved hydronephrosis; Voiding Cystourethrogram showed no VUR.
- 6-month review: Asymptomatic; DTPA proved stable split function (right 53%) and prompt drainage. The renal function tests were normal.
- 12-month telephone follow-up: No recurrence of symptoms or UTIs.

## Discussion

Normal urinary system development hinges on the formation of the ureteric bud and its subsequent interaction with the metanephric mesenchyme^[1,10]^. Errors in this complex developmental choreography can lead to congenital anomalies such as duplex collecting systems and ureteroceles. The Weigert-Meyer law explains that in duplicated systems, the upper pole ureter typically inserts more medially or ectopically within the bladder, predisposing it to obstruction and cystic dilation (ureterocele), while the lower pole is more vulnerable to vesicoureteral reflux (VUR). Ureteroceles are clinically classified as intravesical (orthotopic) or ectopic, depending on their anatomical position relative to the bladder neck. Approximately 20% of adult ureterocele cases are associated with duplex systems, and overall prevalence is estimated at up to 1 in 500 individuals, with a pronounced female predilection and subtle left-sided dominance^[10]^. This highlights the importance of heightened awareness for early detection, even in adults. Adults with ureteroceles often present with recurrent UTIs, hematuria, or flank pain attributable to urinary stasis^[8,11,12]^. Diagnostic imaging begins with ultrasonography, often revealing cystic intravesical lesions. CT urography further characterizes the anatomy, revealing the classic “cobra-head” sign^[8]^. Renography remains indispensable in quantifying differential renal function, critical for surgical planning, especially when nephron-sparing interventions are considered for upper moiety contributions of ≥10%^[11,13]^. A nephron-preserving philosophy is pivotal, particularly in duplex systems, where removal of functional renal tissue should be avoided wherever feasible^[8,13]^. This underscores the importance of taking ureterocele with or without duplex moiety in the differential diagnosis of recurrent UTI, along with the importance of imaging to guide treatment modalities for optimal results. Our patient also presented with a three-month history of intermittent flank pain and a past medical history significant for recurrent culture-positive UTIs, who underwent renal ultrasound followed by CT urography to confirm the diagnosis. A DTPA renogram was conducted to assess the renal function to decide the management approach through TFL decompression over heminephrectomy. The management of duplex kidney depends upon the functioning of the moiety, as heminephrectomy with ureterectomy is preferred for the nonfunctioning kidney, while ureteropyelostomy/uretero-ureterostomy with ureteric common sheath reimplantation has been the standard of care for the functioning moiety^[14]^. In a retrospective study with 3.9-year average post-procedure follow up on 40 pediatric patients with functioning upper moiety, techniques like laparoscopic uretero-ureterostomy compared to common sheath ureteral reimplantation had lesser median hospital stay and comparable non-obstructive, patent anastomosis and no signs of stenotic comprise of the receiving ureter, although the data on outcomes of such procedures on adult is largely lacking^[15]^. The initial endoscopic management of ureteroceles utilized cold-knife and monopolar diathermy incisions. Despite being widely adopted, these techniques were associated with risks such as hemorrhage, incomplete decompression, thermal injury, and significant postoperative reflux rates. Holmium: YAG laser represented a technological advancement, yet carried an inheren ~0.4 mm tissue penetration risk with postoperative reflux reported in 10–30% of cases^[3,6,16]^. Pediatric studies showed reflux rates up to 38%, with one-third of patients requiring reoperations^[6]^. These data emphasize the need for safer, more effective endoscopic alternatives, particularly for delicate congenital anomalies. Thulium Fiber Laser operates at a wavelength of 1940 nm, corresponding to water’s absorption peak, resulting in highly controlled, superficial tissue ablation (~0.2 mm depth). TFL fibers (<150 µm) offer enhanced endoscopic precision and navigation in narrow anatomical spaces. Clinical studies and randomized trials confirm that TFL reduces operative times and tissue trauma, while improving hemostasis and minimizing postoperative complications^[4,5]^. Pediatric ureterocele series using TFL report success rates of approximately 92%, with postoperative reflux reduced to ~8% compared to ~58% for electrocautery^[6,17]^. These findings mark TFL as a superior alternative in congenital urological anomalies. Despite these promising pediatric results, evidence supporting TFL in adult ureterocele management remains anecdotal. Another outcome analysis by Sharma *et al* also demonstrated lower retreatment rates with Holmium compared to electrocautery but still noted edema and VUR post-procedure^[12]^. These observations position TFL as a potentially superior alternative for adult cases, yet large comparative studies remain lacking.

Our patient underwent precise TFL deroofing with dual DJ stenting—an innovative strategy not previously reported in adult duplex ureterocele management. While commonly seen in pediatric reconstructive protocols, its role in adult duplex-system ureteroceles is unprecedented. The dual-stent strategy safeguarded both upper and lower moieties from transient postoperative obstruction, supported healing, and facilitated optimal drainage symmetry.

The total operative time was just 40 minutes with negligible blood loss. At six-month follow-up, hydronephrosis resolved, split renal function was preserved, and no reflux was identified. This success extends favorable pediatric TFL data to adult duplex anatomy, offering compelling evidence for broader adoption.

Despite this positive outcome, our report represents a single experience and lacks direct comparison with Holmium: YAG or other energy sources. Long-term (>12 months) follow-up data are required to rule out rare complications such as ureteric stenosis or late urothelial carcinoma transformation, historically reported in ~1% of cases^[18]^. Future directions should include multicenter prospective registries comparing TFL with Holmium:YAG, optimized dual stenting protocols, and application of genomic precision medicine strategies to personalize surgical decision-making.

## Conclusion

This case describes the successful management of an exceptionally rare clinical scenario—an adult intravesical ureterocele associated with a functioning duplex renal system—using Thulium Fiber Laser (TFL) decompression combined with dual DJ stenting. Adult ureteroceles are uncommon, and while pediatric outcomes with TFL are increasingly documented, reports in adult populations remain sparse. Existing surgical techniques, including cold-knife incision and Holmium: YAG laser, often carry risks of incomplete decompression, postoperative reflux, or collateral tissue damage. In contrast, TFL’s precise energy delivery and shallow penetration allow for controlled incision with minimal complications, as reflected in our favorable clinical outcome of preserved renal function, resolution of hydronephrosis, and absence of reflux. Although adult applications of TFL in ureterocele treatment are not yet well-represented in current literature, this case supports its potential role as a nephron-sparing, minimally invasive alternative. The innovative use of bilateral DJ stenting, adapted from pediatrics surgical strategies, further optimized postoperative drainage and functional preservation.

While larger studies are needed to validate these findings, this report contributes valuable clinical evidence supporting the expansion of TFL use in carefully selected adult ureterocele cases and highlights the need for continued research and standardized guidelines in this evolving area of urologic care.

## Data Availability

The complete data of this research are fully available on request made to the corresponding author.
